# Low-level laser therapy (LLLT) does not reduce subcutaneous adipose tissue by local adipocyte injury but rather by modulation of systemic lipid metabolism

**DOI:** 10.1007/s10103-016-2021-9

**Published:** 2016-07-06

**Authors:** Marek Jankowski, Mariusz Gawrych, Urszula Adamska, Jakub Ciescinski, Zbigniew Serafin, Rafal Czajkowski

**Affiliations:** 1 Chair of Dermatology, Sexually Transmitted Diseases and Immunodermatology, Faculty of Medicine, Nicolaus Copernicus University in Torun, 9 Sklodowskiej-Curie Street, 85-094 Bydgoszcz, Poland; 2 Chair of Radiology and Imaging Diagnostics, Faculty of Medicine, Nicolaus Copernicus University in Torun, 9 Sklodowskiej-Curie Street, 85-094 Bydgoszcz, Poland

**Keywords:** Low-level laser therapy, Subcutaneous adipose tissue, Fat tissue interaction, Body contouring, Laser lipolysis

## Abstract

Low-level laser (light) therapy (LLLT) has been applied recently to body contouring. However the mechanism of LLLT-induced reduction of subcutaneous adipose tissue thickness has not been elucidated and proposed hypotheses are highly controversial. Non-obese volunteers were subject to 650nm LLLT therapy. Each patient received 6 treatments 2-3 days apart to one side of the abdomen. The contralateral side was left untreated and served as control. Subjects’ abdominal adipose tissue thickness was measured by ultrasound imaging at baseline and 2 weeks post-treatment. Our study is to the best of our knowledge, the largest split-abdomen study employing subcutaneous abdominal fat imaging. We could not show a statistically significant reduction of abdominal subcutaneous adipose tissue by LLLT therapy. Paradoxically when the measurements of the loss of fat thickness on treated side was corrected for change in thickness on non treated side, we have observed that in 8 out of 17 patients LLLT increased adipose tissue thickness. In two patients severe side effect occurred as a result of treatment: one patient developed ulceration within appendectomy scar, the other over the posterior superior iliac spine. The paradoxical net increase in subcutaneous fat thickness observed in some of our patients is a rationale against liquefactive and transitory pore models of LLLT-induced adipose tissue reduction. LLLT devices with laser diode panels applied directly on the skin are not as safe as devices with treatment panels separated from the patient’s skin.

## Introduction

Low-level laser therapy (LLLT) is defined as an application of low-irradiancy laser light (1 mW–5 W/cm^2^) where therapeutic results are achieved without photothermal nor photoacoustic effects. LLLT is widely used as a therapeutic modality to promote wound healing and reduce pain, tissue inflammation, and damage [1–4]. More recently, there have been attempts to apply LLLT to treat acne, scars, alopecia, and cellulite. LLLT devices are also marketed for cosmetic use in body contouring and reduction of subcutaneous fat thickness [5–11]. Despite several studies reported a significant reduction of subcutaneous fat thickness induced by LLLT, the exact mechanism by which LLLT acts on fatty tissue has not been elucidated. An original explanation to the phenomenon of fat tissue interaction with laser light, provided by Neira et al., was that laser irradiation permeabilizes cell membranes inducing pores in cell membrane, what results in the passive spillage of lipids into the interstitial space [11]. Alternative hypothesis is that LLLT affects lipid metabolism in adipocytes and/or transport of lipids through cell membrane without affecting its integrity [9].

The aim of this study was to investigate the efficacy and safety of LLLT in reduction of subcutaneous adipose tissue thickness and to verify whether the mode of action on adipose tissue depends on local or systemic mechanisms.

## Methods and procedures

Twenty-four healthy men and women, aged 22 to 55 years with body mass indexes (BMI) below 30 kg/m^2^, were enrolled in the study. The exclusion criteria were obesity, previous liposuction or abdominoplasty, and pregnancy. Subjects agreed to refrain from participating in any other body contouring or weight loss procedures or programs during the course of the study including, but not limited to, over-the-counter and prescription appetite suppressants, diet plans, surgical procedures, and alternative therapies. The study was conducted with the principles of the Declaration of Helsinki and was approved by the ethics committee on research at the Nicolaus Copernicus University.

The subjects’ waist circumference and body weight were measured and photographs were obtained before every treatment. The subjects underwent ultrasound imaging at baseline and 2 weeks post-treatment. Subcutaneous adipose tissue thickness was measured in three points over the left and right rectus abdominis muscle from the dermis-fat interface down to the deep fat-muscle fascia interface.

Each subject received six treatments 2 to 3 days apart with the LLLT device (Lipo Laser, Mimari, Poland) over a 2-week period. The device is composed of six panels measuring 8 by 16 cm each. The 100-mW laser diodes of 650 nm wavelength are distributed in the panel every 4 cm, totaling 8 emitters per panel and 48 emitters in total. The total skin area covered by laser panels was 768 cm^2^, and the surface power density was 9.14 mW/cm^2^. Laser panels were applied directly on the skin for 20 min. Elevated rim of the panel provided 0.5 cm distance of laser diodes from the skin surface. The total laser energy that patients were exposed to during one session was 7.5 J/cm^2^. Only the right side of the abdomen was irradiated; the left side was untreated and served as control.

Clinical assessment of any side effects such as erythema, edema, blistering, hyperpigmentation, hypopigmentation, whitening, purpura, ulceration, and scarring were done before each treatment session and at the follow-up visit 2 weeks after the last treatment.

Statistical analysis was performed using STATISTICA version 12.0 software. Data normality was verified with the Shapiro-Wilk test. Parametric data were expressed as mean ± SD. To analyze the effects of intervention, delta values (Δ) were used obtained from the difference between the post-treatment and baseline values for each variable: Δ variable = post treatment value − baseline value. Comparing the delta values between the groups was performed by *t* test for independent samples, *t* test for dependent samples, and ANOVA for repeated measurements. The net treatment benefit (change) was calculated as a difference between change of fat thickness on treated side and change of thickness on control side (net benefit = (Σ treated side pre-treatment − Σ treated side post treatment) − (Σ control side pre-treatment − Σ control side post treatment)).

## Results

**Fig. 1:**
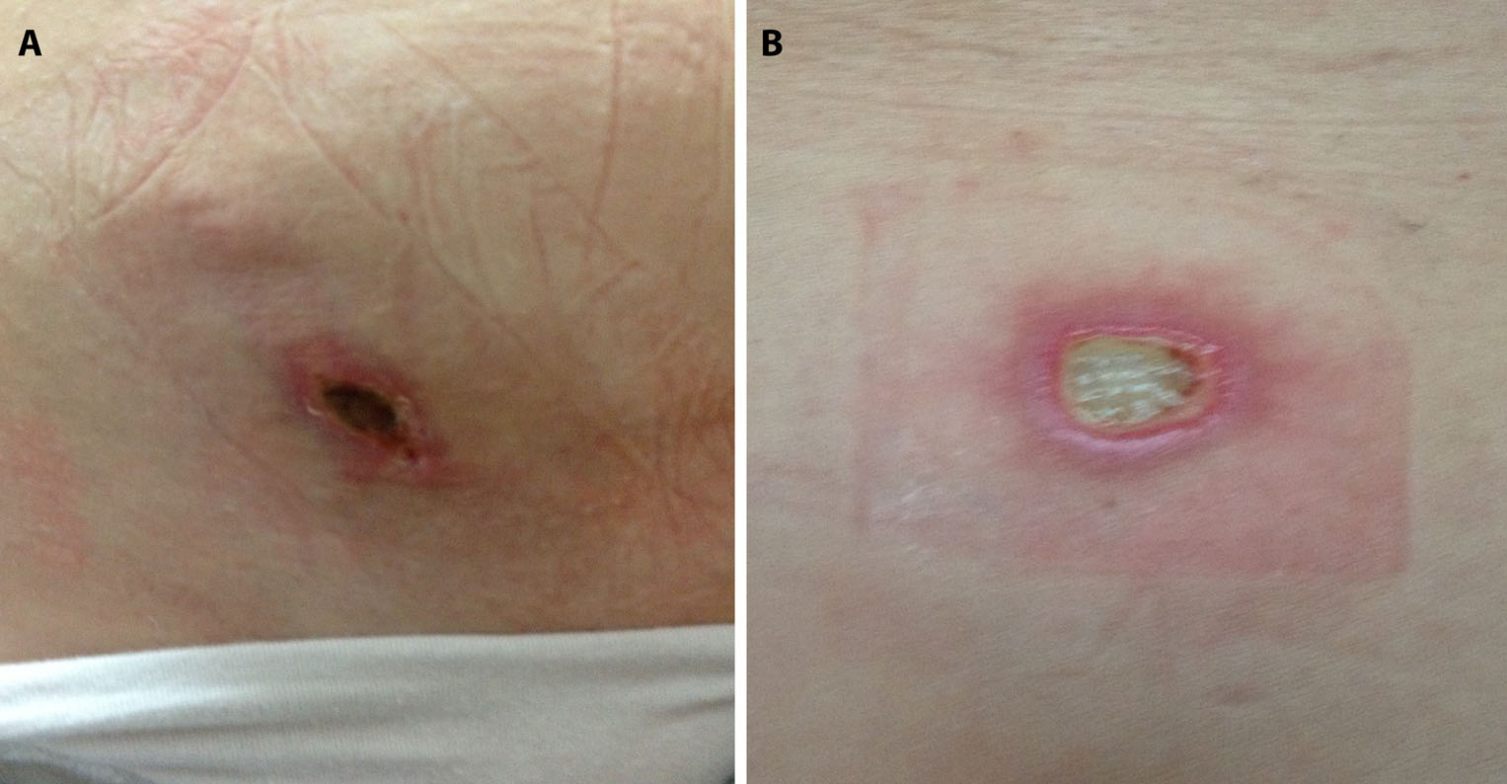
One patient developed ulceration within appendectomy scar, the other over the posterior superior iliac spine

Nineteen subjects completed the study (but two were not available for post-treatment ultrasound evaluation). The reasons for discontinuation of participation in the study was self-assessed lack of benefit (*n* = 3) and side effects (*n* = 2). Erythema lasting more than 24 h after the treatment was reported in four patients, and two of those patients developed ulcerations in irradiated area. One patient developed ulceration within the appendectomy scar, the other over the posterior superior iliac spine (Fig 1).

**Fig. 2:**
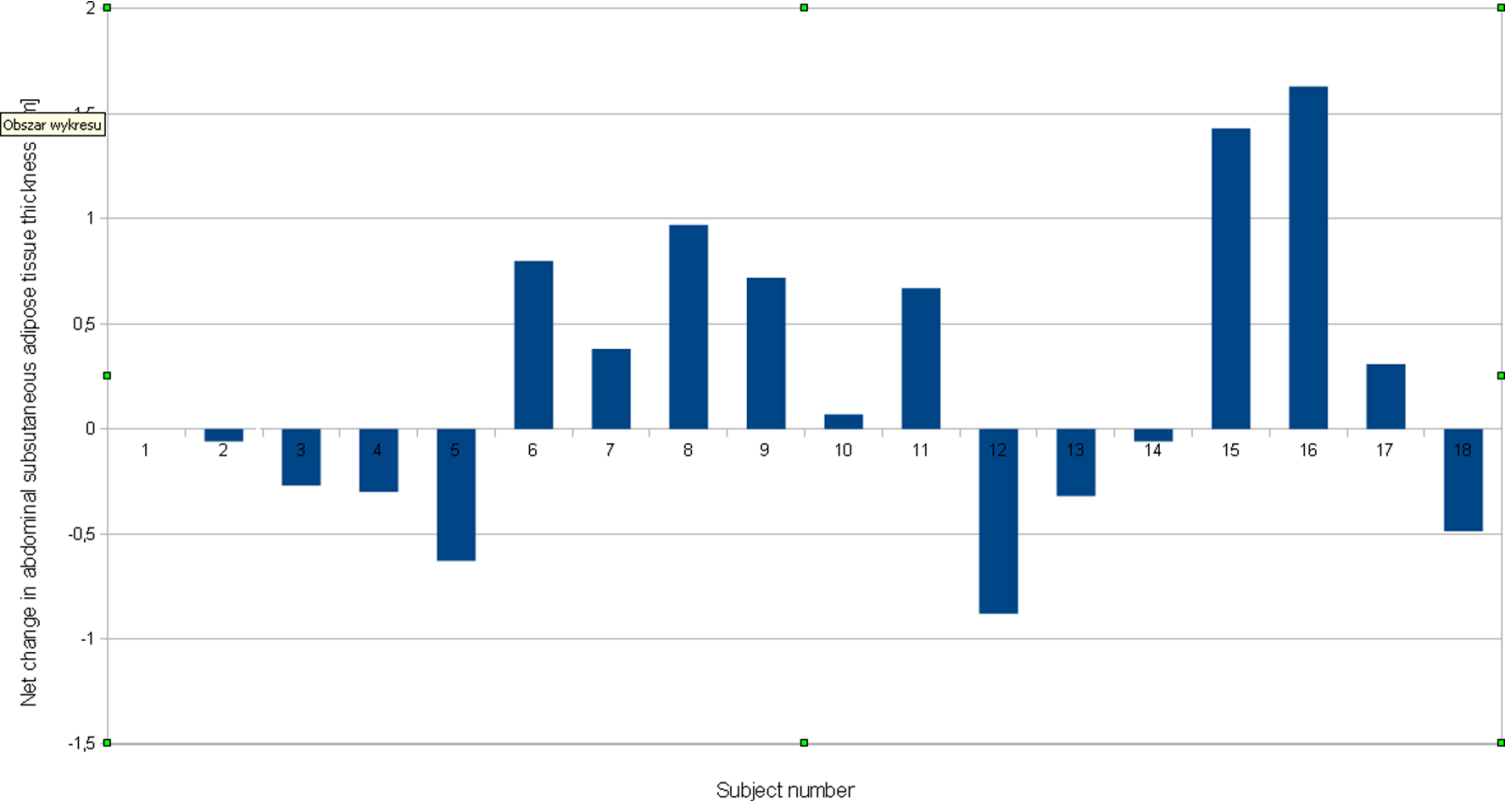
Post-treatment results of individual changes in abdominal fat tissue thickness

The results of anthropometric measurements of the studied population at baseline are summarized in Table 1. Post-treatment results are illustrated as chart of individual changes in abdominal fat tissue thickness in Fig 2 and as descriptive statistics of ultrasound measurements of abdominal adipose tissue thickness in Table 2. Although a trend towards reduction of adipose tissue thickness was observed, the results are statistically not significant (*p* = 0.47). The maximal combined reduction of adipose tissue of 16.3 mm was achieved in patient 15. We have observed a correlation between the weight loss and the reduction of fat thickness on treated (*p* = 0.068) and non-treated (*p* = 0.065) side. Paradoxically, when the measurements of the loss of fat thickness on the treated side was corrected for change in thickness on the non-treated side, we have observed that in 8 out of 17 patients, LLLT increased adipose tissue thickness. In patient 11, the combined increase of adipose tissue thickness totaled to 8.8 mm. No correlation was found between the reduction of adipose tissue thickness and patient’s age, initial weight, initial BMI, initial waist circumference nor sex.

**Table 1:** Anthropometric measurements of the studied population at baseline

Variable	X ± S	V	A_s_	K_u_-3
Age	37.24 ± 13.30	35.72	0.01	−1.91
Body mass (kg)	68.97 ± 13.67	19.82	0.95	1.51
Body mass index (kg/m^2^)	23.60 ± 2.89	12.25	0.36	−0.02
Σ left side of abdomen (cm)	7.24 ± 2.80	38.61	−0.67	−0.24
Σ right side of abdomen (cm)	7.15 ± 2.66	37.28	−0.51	−0.14

*X* arithmetic average, *S* standard deviation, *V* coefficient of variation, *A*_*s*_ coefficient asymmetry, K_u_-3 coefficient of kurtosis

**Table 2:** Descriptive statistics of ultrasound measurements of abdominal adipose tissue thickness

	Side	X ± S [cm]	V	A_s_	K_u_-3
Weight loss (kg)		1.25 ± 1.03	83.59	−0.15	0.38
Waist circumference loss (cm)		1.35 ± 3.10	228.85	0.99	1.05
Combined adipose tissue thickness pre-treatment	Left	7.24 ± 2.80	38.61	−0.67	−0.24
	Right	7.15 ± 2.66	37.28	−0.51	−0.14
Combined adipose tissue thickness post-treatment	Left (control)	7.40 ± 2.70	36.47	−0.64	−0.21
	Right (treated)	7.08 ± 2.45	34.61	−0.32	−0.25

Thickness presented as a sum of three measurements over the rectus abdominis muscle from the dermis-fat interface down to the deep fat-muscle fascia interface. *X* arithmetic average, *S* standard deviation, *V* coefficient of variation, *A*_*s*_ coefficient asymmetry, K_u_-3 coefficient of kurtosis

## Discussion

Compared to previously published studies [7], we have noted a relatively large number of side effects (4 out of 24 patients vs. 0 out of 86). There is an important technical difference between the LLLT device used in our study (Lipolaser, Mimari, Poland) and that of McRae et al.’s (Zerona, Erchonia, USA) that the device used by us has laser-diode panels attached directly to the patient’s skin, whereas one used by McRae et al. has laser diodes operating 15 cm from the patient’s skin. Despite LLLT acting in a non-thermal manner, the waste heat generated by laser diodes may compromise premise of LLLT and cause injury to the patient’s skin if in close contact.

Efficacy of LLLT in reduction of thickness of subcutaneous fat has previously been confirmed by studies involving much larger populations; therefore, one can argue that its failure to show in our study results from insufficient sample. Lack of significance of adipose tissue thickness reduction in our study as compared with strong evidence of efficacy in Jackson’s study (*p* < 0.0001) could be explained by methodological differences, particularly in a much smaller area treated in our study [12].

However, treatment protocols in majority of studies performed to date combined LLLT treatments with, often several months long, intensive aerobic plus resistance training [13–15] or required subjects to utilize a weight loss supplement [7, 16]. Majority of studies designed to verify effects of LLLT alone used circumference measurements as the only estimator of subcutaneous fat layer thickness [8, 9, 12] Estimation of the adipose tissue thickness, especially in the abdominal area based solely on circumference measurements, may be confounded by dietary factors, medical conditions, and medications that cause bloating or swelling. The confounding factors could be removed by the split-abdomen study design and use of objective methods of adipose tissue thickness assessment such as ultrasound or NMR imaging. The only split-abdomen study with ultrasound imaging evaluation of subcutaneous fat layer thickness by Elm et al. [16] enrolled only two patients for LLLT-only arm of the study. Our study is, to the best of our knowledge, the largest split-abdomen study employing subcutaneous abdominal fat imaging.

Our result is clearly discordant with those obtained in a well-designed randomized double-blinded study by Jackson et al. [12] and another large-scale study [10], yet they may be mechanistically reconciled.

Two models of LLLT action on subcutaneous adipose tissue have been proposed in the past. Neira et al. suggested that LLLT disrupts adipocyte cell membranes and allows the lipids to “spill” from the cell [11]. Assuming the liquefactive mode of action of LLLT, one expects prominent local effects with little or no effect in non-treated areas. We have found that in some of our patients, there was a net increase in subcutaneous fat thickness which cannot be reconciled with liquefactive model.

Alternative hypothesis is that LLLT induces triglyceride mobilization from unharmed cells. Caruso-Davis et al. showed increased triglyceride content in supernatants of LLLT-treated primary adipocytes while the cells themselves showed decreased calcein staining post-treatment compared to non-treated cells [9]. The viability of treated and non-treated cells, as determined by the propidium iodide assay, was similar; hence, Caruso-Davis et al. suggested that this proves formation of transitory pores in the cell membrane. However, in our opinion, upregulation of multidrug resistance protein 1, responsible for calcein extrusion from the cells, would present with the same results. Again, the net increase in subcutaneous fat thickness observed by us in some of our patients is difficult to be reconciled with transitory pore model.

In a study by Jackson et al. [12], significant circumferential reduction in non-treated areas was reported what could suggest that LLLT affects systemic lipid metabolism, possibly involving an intermediate acting in an autocrine/paracrine manner. Animal studies seem to support this hypothesis and further corroborate our results. Aquino et al. showed that LLLT significantly decreased relative mass of fat tissue in trained animals while it tended to increase body weight and fat content in sedentary animals [17]. The paradoxical positive effect of LLLT in some of our patients and negative in others could result from differences in the patients’ physical exercise routines; unfortunately, our study did not measure this variable.

A large body of evidence suggests that vascular oxidative stress induces obesity and metabolic syndrome [18] while oxidative stress in adipose tissue not only correlates with insulin resistance but is also causative in its development [19]. Moreover, oxidative stress in adipose tissue decreases adiponectin secretion [20] resulting in a decrease of adiponectin-induced energy expenditure associated with protein uncoupling [21]. LLLT has been shown to reduce oxidative stress in neural and muscular tissue [3, 22]; however, as to our knowledge, the effects of LLLT on adipocyte oxidative stress levels have not been studied. Further studies are necessary to elucidate this problem. It is tempting to speculate that LLLT-induced reduction of adipose tissue thickness results from decreased oxidative stress in adipocytes and consequently increased adiponectin secretion and decreased insulin resistance. Under this model, the combination of LLLT and aerobic exercise is crucial for its efficacy in the reduction of subcutaneous adipose tissue thickness.
